# Symbiotic flagellate protists as cryptic drivers of adaptation and invasiveness of the subterranean termite *Reticulitermes grassei* Clément

**DOI:** 10.1002/ece3.3819

**Published:** 2018-05-10

**Authors:** Sónia Duarte, Tânia Nobre, Paulo A. V. Borges, Lina Nunes

**Affiliations:** ^1^ Structures Department LNEC Lisbon Portugal; ^2^ Faculty of Agrarian and Environmental Sciences cE3c – Centre for Ecology, Evolution and Environmental Changes/Azorean Biodiversity Group University of the Azores Azores Portugal; ^3^ Laboratory of Entomology ICAAM ‐ Instituto de Ciências Agrárias e Ambientais Mediterrânicas University of Évora Évora Portugal

**Keywords:** adaptation, aggressiveness, biodiversity, invasive species, rural environment, subterranean termite, symbiotic flagellate protists, urban environment

## Abstract

Changes in flagellate protist communities of subterranean termite *Reticulitermes grassei* across different locations were evaluated following four predictions: (i) Rural endemic (Portugal mainland) termite populations will exhibit high diversity of symbionts; (ii) invasive urban populations (Horta city, Faial island, Azores), on the contrary, will exhibit lower diversity of symbionts, showing high similarity of symbiont assemblages through environmental filtering; (iii) recent historical colonization of isolated regions—as the case of islands—will imply a loss of symbiont diversity; and (iv) island isolation will trigger a change in colony breeding structure toward a less aggressive behavior. Symbiont flagellate protist communities were morphologically identified, and species richness and relative abundances, as well as biodiversity indices, were used to compare symbiotic communities in colonies from urban and rural environments and between island invasive and mainland endemic populations. To evaluate prediction on the impact of isolation (iv), aggression tests were performed among termites comprising island invasive and mainland endemic populations. A core group of flagellates and secondary facultative symbionts was identified. Termites from rural environments showed, in the majority of observed colonies, more diverse and abundant protist communities, probably confirming prediction (i). Corroborating prediction (ii), the two least diverse communities belong to termites captured inside urban areas. The Azorean invasive termite colonies had more diverse protist communities than expected and prediction (iii) which was not verified within this study. Termites from mainland populations showed a high level of aggressiveness between neighboring colonies, in contrast to the invasive colonies from Horta city, which were not aggressive to neighbors according to prediction (iv). The symbiotic flagellate community of *R. grassei* showed the ability to change in a way that might be consistent with adaptation to available conditions, possibly contributing to optimization of the colonization of new habitats and spreading of its distribution area, highlighting *R. grassei* potential as an invasive species.

## 1 INTRODUCTION

The last decades’ rural exodus created an expansion of urban areas and a complete change in rural landscapes (e.g., Brazil, Lapola et al., 2014). Species distribution is directly affected by the changes in rural and urban landscapes. In urban areas, an island‐like effect could be considered, arising from the patchiness of available habitats which favor the isolation of species populations; however, contrary to common expectations, species diversity is not necessarily lower in northern European urban areas (Knapp, Kühn, Mosbrugger, & Klotz, 2008; Knop, 2016). On one side, the biotic homogenization effects due to urbanization processes are visible among some indicator taxa, usually resulting in lower species richness, on the other side, the installation of non‐native species can counteract this effect, increasing species richness (Knop, 2016). Urbanization process often leads to the loss of native species but also to the recruitment of other (new) species. Invasive and synanthropic species are usually highly associated with urban settings (Shochat et al., 2010). Termites are frequently dominant in tropical and temperate rural areas, and are also common in urban areas; despite their importance in diverse ecosystems, they may be considered severe pests of wood in service, and as an agricultural and forestry pest (Constantino, 2002; Rouland‐Lefèvre, 2011). Subterranean termites represent approximately 80% of the economically important species in this context (Nobre & Nunes, 2007; Rust & Su, 2012). The continuous growth of human population leading to urbanization and the effects of climate change may further change termite species distribution patterns (Ewart, Nunes, de Troya, & Kutnik, 2016). Natural distribution ranges of native termites may tend to adapt to the new created conditions, and eventual human‐mediated introduction events might increase.

There are some examples of subterranean termites with lower genetic variability in their invasive range: *Reticulitermes urbis* (Bagnères, Uva and Clément; Blattodea: Isoptera: Rhinotermitidae), native from the Balkan Peninsula, in France and Italy (Leniaud, Dedeine, Pichon, Dupont, & Bagnères, 2010); R*eticulitermes flavipes* (Kollar), native from United States, in France; and *Reticulitermes grassei* Clément, native from the Iberian Peninsula and Atlantic coast of France, in the UK, although this last example may have been influenced by the genetic markers used in the study (Jenkins, Dean, Verkerk, & Forschler, 2001; Kutnik, Uva, Brinkworth, & Bagnères, 2004; Leniaud et al., 2010; Perdereau et al., 2015). However, social insects are known to have some robustness to the loss of genetic diversity during introduction events, which may contribute to their high invasiveness capacity, as they represent a quarter of the 100 worst invasive insect species (Lowe, Browne, Boudjelas, & De Poorter, 2000). Oceanic islands are especially sensitive to disturbances from invasive alien species, due to their high endemism and isolation (Reaser et al., 2007).

The role of symbionts within invasive strategies is usually overlooked; however, a holobiotic approach that considers host and symbionts as an evolutionary unit is needed, as each symbiont carries new genes, with the potential to affect host invasiveness ability and host ecology (Cass et al., 2016; Kikuchi et al., 2012; Rosenberg & Zilber‐Rosenberg, 2013).

The European subterranean termite *R. grassei* is native to the Iberian Peninsula and the Atlantic coast of France (Kutnik et al., 2004). An invasive population of *R. grassei* was identified in the United Kingdom, apparently representing a typical genetic bottleneck in an exotic setting (Jenkins et al., 2001), in contrast to the heterogeneity found among colonies in native areas (Kutnik et al., 2004). Another invasive population belonging to this species of termite was identified in Horta city (Faial island in the Azores Archipelago) in the early 2000s infesting a coastal southern part of the city, and since then, it was identified in other structures in the city (Ferreira et al., 2013). For distribution maps of *R. grassei* in Europe, see, for example, Austin, Szalanski, Uva, Bagnères, and Kence (2002); for Azorean island distribution, see Ferreira et al. (2013).

Subterranean termites live in symbiosis with flagellate protists and bacteria, harbored in the dilated portion of the hindgut, the paunch. In this tripartite lignocellulolytic system the termite contributes with endogenous cellulases and mechanical processing, flagellate protists phagocyte the wood particles and digest them, and prokaryotes have, among others, an important role in maintaining the physical–chemical equilibrium inside the termite hindgut (Peterson, Stewart, & Scharf, 2015; Xie et al., 2012). A significant part of cellulose digestion process is performed by flagellated protists, which are part of the unicellular eukaryotes belonging to two separate lineages: the order Oxymonadida (Phylum Preaxostyla) and the Phylum Parabasalia (Adl et al., 2012; Brugerolle, 1991; Čepička, Hampl, & Kulda, 2010).

Geographical variation of symbiotic flagellate protists was studied for *Hodotermopsis sjoestedti* Holmgren and *Reticulitermes* genus, in Japan (Kitade, Hayashi, Takatsuto, & Matsumoto, 2012; Kitade & Matsumoto, 1993; respectively); however, the possible variables involved in the observed geographical variation were not investigated. The presence of each flagellate protist species in different termite colonies sampled was usually very high, reinforcing the hypotheses that flagellate protists are strongly associated with their hosts and that the main driving force shaping flagellate protist communities living inside termites is host phylogeny (Tai et al., 2015).

This study focuses on symbionts of the termite *R. grassei* and aims to investigate the changes in flagellate protist communities of the termite across different locations within limited geographical variation: colonies from urban and rural environments and island invasive and mainland endemic populations. The colonies were compared using not only symbiont species richness and abundances, but also other biodiversity indices. For further characterization of island invasive and mainland endemic termite populations, aggression tests among termites’ populations and colonies were also performed. We predict that: (i) rural native termite populations will exhibit high diversity of symbionts; (ii) on the contrary, urban invasive populations, with putatively lower environmental diversity, will exhibit lower diversity of symbionts and could increase the similarity of symbiont assemblages through environmental filtering (i.e., biotic homogenization); (iii) the colonization of isolated regions as is the case of islands will imply a loss of diversity of symbionts; and (iv) island isolation will trigger a change in colony breeding structure toward a less aggressive behavior.

## 2 METHODS

### 2.1 Geographic variation in symbiont species composition, abundance, and richness

**Figure 1:**
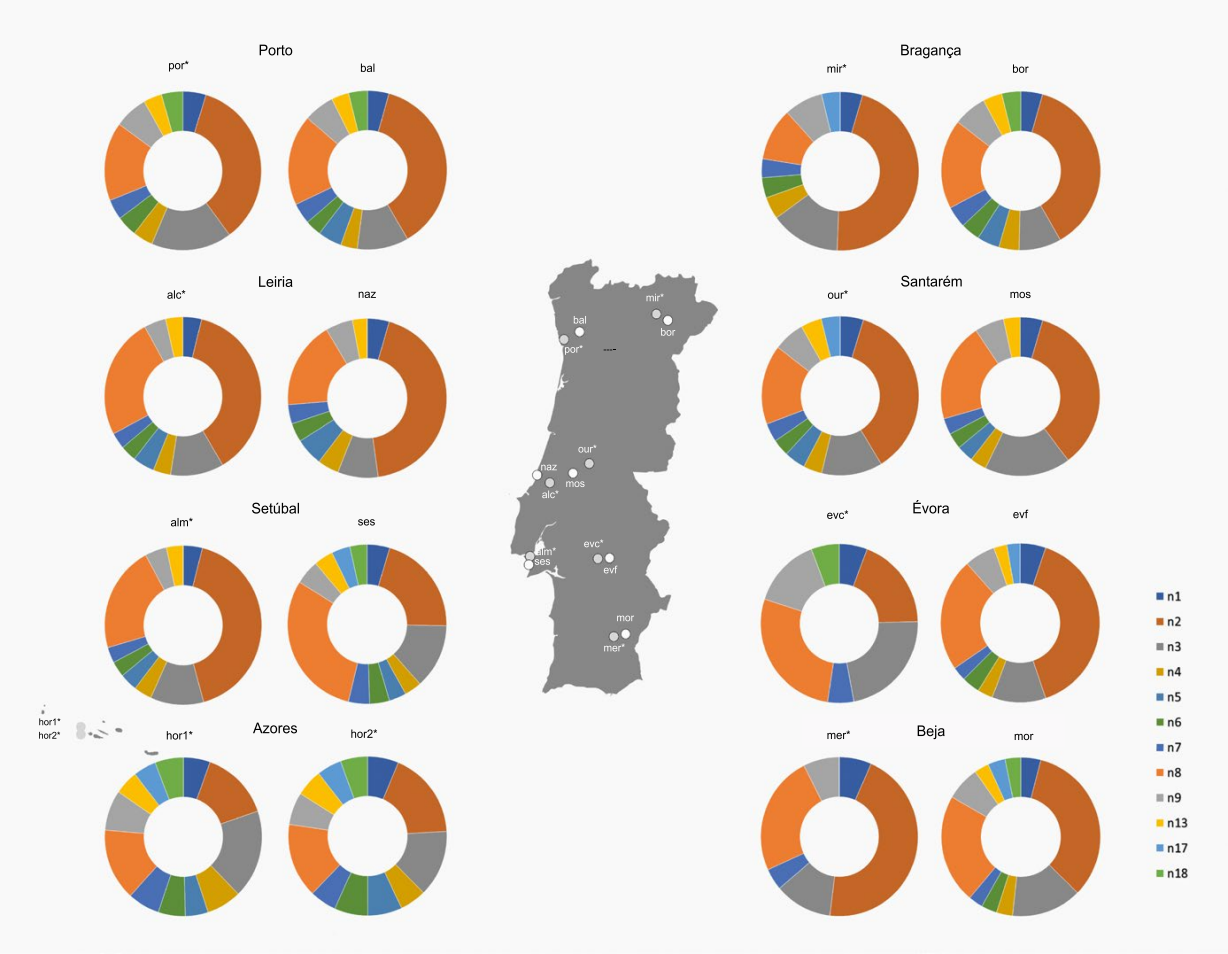
Doughnut charts for flagellate protist community (morphotypes: n1, n2, n3, n4, n5, n6, n7, n8, n9, n13, n17, and n18) of termites captured in 16 locations shown on the map of continental Portugal, with two additional sites from the Azores (light gray circles—urban environment; white circles—forest environment): Azores—Faial island: two locations inside Horta city (hor1 and hor2); Bragança: Mirandela (mir) and Bornes (bor); Beja: Mértola (mer) and Moreanes (Mor); Évora: Évora city (evc) and Évora forest (evf); Leiria: Alcobaça (alc) and Nazaré (naz); Porto: Porto (por) and Baltar (bal); Santarém: Ourém (our) and Porto de Mós (mos); Setúbal: Almada (alm) and Sesimbra (ses). Note: names followed by an asterisk refer to an urban environment opposing to the remaining names which refer to a rural environment. Portugal map from Portugal—Single Color by FreeVectorMaps.com

Sixteen different colony samples of *Reticulitermes grassei* termites were captured between April and July 2015, fourteen from seven regions covering continental Portugal and two from non‐native colonies in the Azorean island of Faial (Horta; Figure 1).

In each region, one colony from an urban environment and one colony from a rural environment were sampled (Table 1), except for the non‐native range, in which both colonies were captured in the city of Horta. A systematic search of other subterranean termite colonies was performed in target zones within Faial Island different natural habitats and towns. No other colonies were found leading to the conclusion that, probably, these termites are so far only established in a few neighborhoods of the city of Horta.

**Table 1:** Sampling points and characteristics (district, coordinates, environment, and type of conditions) and of the substrate in which the termites were foraging when captured

	Code	Coordinates	Environment	Type	Substrate
Azores (AZ)					
Horta	hor1*	38°31′43′′N 28°37′40′′W	Urban	Backyard	Pallet board
Horta	hor2*	38°31′41′′N 28°37′42′′W	Urban	House	Timber beam
Bragança (BA)					
Mirandela	mir*	41°28′49′′N 7°12′7′′W	Urban	Public garden	Fallen branch
Bornes	bor	41°27′53′′N 6°59′50′′W	Rural	Trees	Fallen branch
Beja (BE)					
Mértola	mer*	37°38′11′′N 7°39′56′′W	Urban	House	Door frame
Moreanes	mor	37°39′21.′′N 7°33′12′′W	Rural	Trees	Fallen branch
Évora (EV)					
Évora (city)	evc*	38°56′86′′N 7°92′54′′W	Urban	House	Pallet board
Évora (rural)	evf	38°59′83′′N 7°94′5′′W	Rural	Trees	Fallen branch
Leiria (LE)					
Alcobaça	alc*	39°50′51′′N 8°50′6′′W	Urban	Public garden	Fallen branch
Nazaré	naz	39°35′30′′N 9°2′17′′W	Rural	Trees	Fallen branch
Porto (PO)					
Porto	por*	41°9′13′′N 8°34′5′′W	Urban	Public garden	Fallen branch
Baltar	bal	41°12′1′′N 8°24′7′′W	Rural	Trees	Fallen branch
Santarém (SA)					
Ourém	our*	36°64′30′′N 8°35′35′′W	Urban	Public garden	Fallen branch
Porto de Mós	mos	39°33′1′′N 8°58′41′′W	Rural	Trees	Fallen branch
Setúbal (SE)					
Almada	alm*	38°39′33′′N 9°10′5′′W	Urban	Public garden	Fallen branch
Sesimbra	ses	38°33′17′′N 9°8′92′′W	Rural	Trees	Fallen branch

Termites were identified to species by the authors according to morphological characters (Clément et al., 2001). Molecular identification was also performed, with results corroborating the termite identification to the species *R. grassei* (S. Duarte et al., *unpublished*).

Twenty worker termites in the 4th instar of development per sampling point were analyzed individually in terms of symbiont protists diversity and abundance by direct observation under a microscope. For quantification of flagellate protists, a hemocytometer was used, in accordance with a previously described method (Duarte, Duarte, Borges, & Nunes, 2017), with an adaptation in the calculations for the number of flagellate protists per microliter of hindgut fluid, which were estimated to the observed area of the hemocytometer following the formula:

$$C=(N\times 160,000/S_{q}\times D_{f})/1,000$$

*N* is the number of cells visualized, *S*_*q*_ is the number of squares, and *D*_*f*_ is the dilution factor.

Flagellate protists were identified according with species, or major taxa, descriptions (Brugerolle, 2006; Brugerolle & Bordereau, 2006; Brugerolle & Lee, 2000; Leidy, 1877; Lewis & Forschler, 2006), based on morphological characters and separated to 12 morphotypes, some of them resembling flagellate protists species already identified within termite hindguts (Table 2). Further research using molecular markers is being pursued to establish robust identification based on both morphological and molecular data.

**Table 2:** Flagellate protist identification to morphotypes (n1–n9, n13, n17, and n18) based on morphological characters

Morphotype	Phylum	Class	Order	Family	Genus	Species
n18	Parabasalia					
n17		Trychonymphea	Trichonymphida	Trichonymphidae		
n1					*Trichonympha*	*T. agilis*
n9		Spirotrichonymphea	Spirotrichonymphida	Holomastigotoididae	*Spirotrichonympha*	*S. flagellata*
n4					*Holomastigotes*	*H. elongatum*
n8					*Microjoenia*	*M. hexamitoides*
n13		Trichomonadea	Honigbergiellida	Tricercomitidae	*Hexamastix*	
n6		Hypotrichomonadea	Hypotrichomonadida	Hypotrichomonadidae		
n2	Preaxostyla		Oxymonadida		*Pyrsonympha* sp.1	
n7					*Pyrsonympha* sp.2	
n3					*Dinenympha*	*D. gracilis*
n5						*D. fimbriata*

Analyses of variance were performed among termite colonies from the same district and all over mainland Portugal, to check the null hypothesis (H0: flagellate protist community diversity and abundance are not significantly different among termite colonies from urban and forest environments; *p* < .05). The independent variables were the rural/urban habitats and the morphotypes identified, with abundances of flagellate protists as the dependent variable. The Tukey honest significant differences post hoc test was applied, for *p* < .05. These analyses were made using package “stats” of R (R Development Core Team, 2014; R Studio Team, 2015).

A dissimilarity matrix calculation, based on Bray–Curtis index, was performed, using the “vegan” package of R (Oksanen et al., 2016), to look for similarities between termite flagellate protist communities. The Bray–Curtis metric was chosen because it considers not only the presence/absence data, but also the abundances of each identified morphotype.

To quantify the effect of geographical location and local factors in the flagellate protist communities, a variation partitioning was performed. Using a series of redundancy analyses, the data were grouped as such: the dependent variables (morphotypes and abundances of each morphotype for each location); the geographical variables (algebraic calculation based on geographical coordinates of each location); and the local variables (whether urban or rural environments; and the type of substrate termites were captured from, see Table 1).

### 2.2 Community diversity indices

The following diversity metrics of termite symbiont community structure were calculated for each colony according to the Hill series strategy: total abundance (N), linked to community biomass; species richness (S), which may act as a surrogate of functional diversity; Shannon–Wiener (H′), a measure of community complexity; Simpson (D) and Berger–Parker (d), both measure dominance; and evenness (E), which measures the equitability of species abundances (Magurran, 2003).

### 2.3 Aggression tests

Termites were collected, as described before, and the aggression tests were performed in the laboratory, <2 weeks after the collection. Collection occurred between March and April 2016, in three locations: Lagoa de Albufeira (Setúbal region; seven colonies); Ferreira do Zêzere (Santarém region; two colonies); and Horta city (in Faial Island of the Azores; five colonies). Distances between locations are as follows: approximately 150 km between Ferreira and Lagoa, and Horta is approximately 1800 km distant from the other two locations. Within each location, termites of different colonies were captured with an estimated distance of 50 to 70 m. The aggressive testing protocol developed was adapted based on protocols from different authors (Huang, Guan, Shen, Hu, & Zhu, 2012; Husseneder & Grace, 2001; Jmhasly & Leuthold, 1999; Kaib et al., 2004; Perdereau, Dedeine, Christidès, Dupont, & Bagnères, 2011). Five termite workers belonging to the same colony were put inside a petri dish with moistened filter paper, together with other five termite workers belonging to different colonies (intercolonial testing) or to the same colony (intracolonial testing—as control experiment). The termite behavior considered was biting, with bodily contact, and use of the mandibles. Termites were observed each minute until 30 min and onward each five minutes until 120 min, in a total of 48 observations, and the number of termites involved in aggressive behaviors was recorded in each observation. An aggression index (*A*_*i*_) was calculated, ranging from zero (no termites showing aggressive behavior) to ten (all termites involved in aggressive behavior; Husseneder & Grace, 2001). The number of surviving termites was recorded at the end of observation time (120 min) and 24 hr later.

## 3 RESULTS

### 3.1 Geographic variation in symbiont species composition, abundance, and richness

All sixteen colonies had six morphotypes in common (n1, n2, n3, n7, n8, n9; Figure 1; for the number of protists, see Appendix S1). Overall analysis of urban versus rural environment indicates a significant difference between these two types of habitats (*p* < .01). Two morphotypes were also considered to have significantly different abundances when comparing urban and rural habitats: n3 and n8 (*p* < .01). Regarding the comparison of urban and rural protist species richness and abundance within the same district, the results were as follows: Leiria did not show significant differences between the populations from urban and rural environments (*F* = 0.3; *p* = .568). All the other districts showed significant differences between urban and rural captured termites in terms of their flagellate protist communities’ species richness and abundance, with rural locations usually having higher richness and abundance of symbionts, except in Santarém, in which urban termites’ symbiotic fauna showed a higher species richness, but lower abundance in Bragança (*F* = 10.1; *p* = .002), Porto (*F* = 17.3; *p* < .001), Santarém (*F* = 10.8; *p* = .001), Setúbal (*F* = 9.4; *p* = .002), Évora (*F* = 95.2; *p* < .001), Beja (*F* = 34.4; *p* < .001). The two populations from Horta did not show significant differences (*F* = 0.3; *p* = .633).

**Figure 2:**
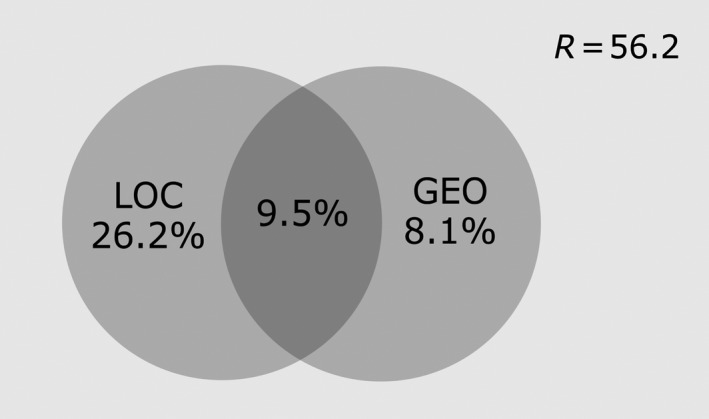
Venn diagram resulting from variation partitioning of geographical variables (GEO) and local conditions (LOC). R represents the residual variation not explained by this model. The figure is not drawn to scale

**Figure 3:**
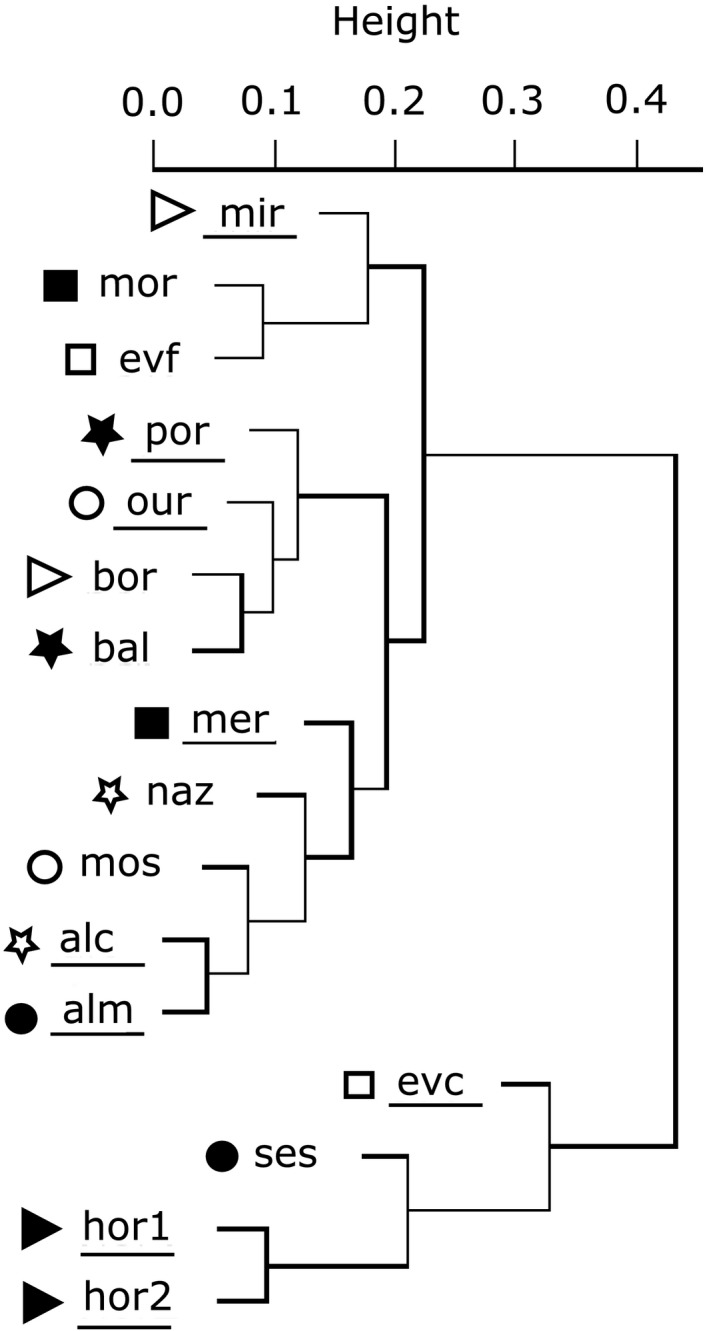
Cluster dendrogram based on Bray–Curtis dissimilarity index among flagellate protist communities of each location (locations belonging to the same geographical region are preceded by the same symbol): Azores—Faial island: two locations inside Horta city (hor1 and hor2); Bragança: Mirandela (mir) and Bornes (bor); Beja: Mértola (mer) and Moreanes (Mor); Évora: Évora city (evc) and Évora forest (evf); Leiria: Alcobaça (alc) and Nazaré (naz); Porto: Porto (por) and Baltar (bal); Santarém: Ourém (our) and Porto de Mós (mos); Setúbal: Almada (alm) and Sesimbra (ses). Note: underlined names refer to an urban environment opposing to the remaining names which refer to a rural environment

For variation partitioning, the results obtained are represented in the Venn diagram (Figure 2). Only 43.8% of the total variation observed within the data is explained by the variables included in this study (56.2% represents the residual variation (R), which is not explained by this model), with local conditions (LOC) and geographical variables (GEO) explaining 26.2% and 8.1% of the variation observed, respectively. The results of the dissimilarity matrix are displayed in the dendrogram (Figure 3). It is possible to discriminate some geographical groups (the north clade [por, our, bor, and bal], the two south forest populations [mor and evf], the country south and center clade [mer, naz, mos, alc, and alm], and the two Azorean populations [hor1 and hor2]), grouped together with ses and evc, although the dissimilarity index between the Azorean populations and evc is high (Figure 3).

### 3.2 Community diversity indices

**Figure 4:**
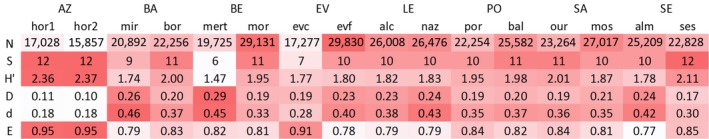
Community diversity indices, abundance (N), species richness (S), Shannon–Wiener (H’), Simpson (D), Berger–Parker (d), and equitability (E), of the termite flagellate protist communities from the studied regions and locations [Azores—Faial island (AZ): two locations inside Horta city (hor1 and hor2); Bragança region (BA): Mirandela (mir) and Bornes (bor); Beja region (BE): Mértola (mer) and Moreanes (Mor); Évora region (EV): Évora city (evc) and Évora forest (evf); Leiria region (LE): Alcobaça (alc) and Nazaré (naz); Porto region (PO): Porto (por) and Baltar (bal); Santarém region: Ourém (our) and Porto de Mós (mos); Setúbal region (SE): Almada (alm) and Sesimbra (ses)]. Note: Inside each region of Portugal mainland, the first location refers to an urban environment and the second location refers to a rural environment

Generally, within each region, the termite populations from rural environments showed higher species richness when comparing to termite flagellate protists from urban environments, except for Leiria (LE) and Santarém (SA; equivalent; and rural showing higher abundance but lower species richness, respectively; Figure 4). In terms of flagellate protists abundance, the tendency was similar, with rural populations showing higher abundances, except for Setúbal (SE), in which the opposite was observed, and Leiria, in which the abundances of both environments studied were quite similar. Both Azorean termite populations seem to be clearly different from mainland populations in terms of their flagellate protist community structure, as both island populations had lower abundance, higher species richness, higher Shannon–Wiener, and higher equitability indices. On the contrary, their dominance indices indicate a lower dominance effect.

### 3.3 Aggression tests

The aggressiveness index for intracolonial tests was zero for all colonies studied, as expected (Table 3). For intercolonial tests, the results ranged from aggressiveness index of zero from termites belonging to different colonies in the Azores, to the high aggressiveness indices among neighboring colonies of termites from the two locations in the mainland. Colonies from different locations displayed lower aggressiveness (comparing to neighbor colonies) and higher survival rates after 24 hr.

**Table 3:** Aggressiveness indices (*A*_*i*_ and *SE*), survival rate at 120 min (S120) and 24 hr (S24) after the beginning of the aggression tests (and *SE*), and the number of trials performed (*n*)

	*A*_*i*_	S120	S24	*n*
Ferreira do Zêzere (F)				
Intracolonial	0.00 ± 0.00	100.0 ± 0.0	100.0 ± 0.0	4
Intercolonial	1.06 ± 0.09	55.0 ± 7.1	10.0 ± 14.1	2
Azores/Horta (A)				
Intracolonial	0.00 ± 0.00	100.0 ± 0.0	100.0 ± 0.0	6
Intercolonial	0.00 ± 0.00	100.0 ± 0.0	100.0 ± 0.0	16
Lagoa de Albufeira (L)				
Intracolonial	0.00 ± 0.00	100.0 ± 0.0	100.0 ± 0.0	8
Intercolonial	1.31 ± 0.48	25.4 ± 12.0	11.5 ± 12.8	13
FA				
Intercolonial	0.47 ± 0.44	71.7 ± 26.0	53.3 ± 34.5	8
FL				
Intercolonial	0.68 ± 0.44	58.3 ± 21.6	30.0 ± 16.3	4
LA				
Intercolonial	0.76 ± 0.58	68.6 ± 22.7	28.6 ± 30.8	7

F, Ferreira do Zêzere; A, Azores/Horta; L, Lagoa de Albufeira; FA, termites from Ferreira do Zêzere and Azores were tested; FL, termites from Ferreira do Zêzere and Lagoa de Albufeira were tested; FA, termites from Lagoa de Albufeira and Azores were tested.

## 4 DISCUSSION

The group of six flagellate protist morphotypes common to all termite colonies in this study may constitute a “core” symbiotic protist microbiome which possibly covers the main functions performed by this group of symbionts within the *R. grassei* termite holobiont.

The remaining morphotypes may constitute facultative symbionts, which, although not being essential for host survival, may provide important benefits, acting like a horizontal gene pool that potentially influences host invasiveness and ecology (Henry et al., 2013; Kikuchi et al., 2012). The uniqueness of termite colony symbiotic communities has recently been shown for *R. flavipes* (Scharf et al., 2017).

In accordance with the first prediction, termites from rural environments showed, in the majority of the situations, more diverse and abundant symbiotic flagellate protist communities. The two least diverse communities belonged to termites captured inside cities, corroborating the second prediction: One sample was captured on inside carpentry (Mértola city), and the other was captured in a wooden pallet stored in a backyard (Évora city). The possible effect of environmental filtering in urban habitats (Aronson et al., 2016) may influence the symbiotic flagellate communities living inside termites. For example, the absence of morphotype n5 from southern Portugal (Beja and Évora districts) and from Porto and Mirandela cities colonies may indicate the influence of environmental filtering. For the flagellate protist communities, the factors acting as sources of variation among termite colonies should be similar to symbiotic bacteria, except that the effect of termite host phylogeny is considered to be more influential than for bacteria (Tai et al., 2015). The genetic divergence among termite colonies may constitute another explanation for the absence of morphotype n5 in the southern colonies. Nobre, Nunes, Eggleton, and Bignell (2006) have shown, in mainland Portugal, a geographic component of the *R. grassei* genetic polymorphism (based on COII marker) and that discrete termite populations have thus differentiated in situ. As stated by Nobre et al. (2006), considering natural forest termite population genetics, the distribution of *Reticulitermes* populations in Portugal is mainly the result of the natural population dynamics without an overall anthropogenic effect. In the present study, which considered not only rural but also urban areas, the same conclusion seems to apply regarding the flagellate protist communities. Nonsharing of symbionts could arise from the limited current levels of host genetic exchange (Nobre et al., 2006).

The residual variation of the variation partitioning analysis, which remained unexplained with the batch of variables available within this work, clearly suggests other factors as influencing the flagellate protist communities associated with habitat specificities, as the rural and urban different conditions in terms of, for example, natural food availability and soil properties. A geographical pattern is suggested in the dissimilarity analysis, which allowed to discriminate some geographical groups, although, for example, the three most diverse flagellate protist communities are grouped together regardless of their geographical origin (hor1, hor2, and ses; see Figure 3); however, the local factors seem to have a major role on the determination of the flagellate protist community variability and/or eventually natural variation should also be considered. The termite diet is one of such influential local factors on the flagellate protist communities of *R. grassei* belonging to the same colony (Duarte, Duarte et al., 2017). However, regarding termites in their natural range, it is very difficult to identify or specify the actual food ingested whatever the type of sources addressed during the observation of foraging; such ingested materials probably comprise a subset of the available cellulolytic material. Using the information on the wood species in which a certain colony was found may be potentially misleading, especially bearing in mind that workers of the same colony exchange gut contents through trophallaxis. The geographical variation observed shall be strongly influenced by environmental conditions (temperature, soil moisture, or food availability), and this requires termite colony plasticity in terms of different social organization and different foraging strategies and the consequent adaptation of their flagellate protist communities (Vargo et al., 2013).

Even though the genetic diversity of the flagellate protists was not accessed within this study, the morphotyping seems to be representative regarding flagellate protist functional diversity. This means that, for example, different species belonging to the *Trichonympha* genus may be present, but the function they are performing inside the termite hindgut is probably equivalent. This hypothesis may be arguable, as studies regarding the functional role of flagellate protists are still scarce, and in addition, the taxonomy of flagellate protists of termites is still hampered by the difficulties of managing molecular classification of this group, involving single‐cell isolation from the termite hindgut (e.g., Duarte, Nunes, Borges, Fossdal, & Nobre, 2017; Harper, Gile, James, Carpenter, & Keeling, 2009). Additionally, the existence of cryptic flagellate species was already detected in previous studies (e.g., Harper et al., 2009).

Contradicting our third prediction, the Azorean invasive termite colonies studied showed more diverse and less numerous flagellate protist communities. The ranges of island diversity indices were different from the endemic mainland populations of termites, showing a more complex and balanced community, possibly performing a wide variety of functions, and less influenced by one or few species in detriment of others, which may give them an adaptive advantage. The hindguts of termites belonging to the same colony may function as islands due to the difficulty of new flagellates colonizing successfully. Indeed, termite symbiotic fauna is known to be extremely closed to external agents, as the symbionts may activate defensive mechanisms toward external agents (Chouvenc & Su, 2012). Furthermore, termites tend to have a protective role in terms of their symbiotic fauna, avoiding the free exchange of hindgut fluids among different species, either by trophallaxis or by the consumption of dead bodies (Sun, Kenneth, & Zhou, 2013). This phenomenon indicates a limited dispersion capacity and the difficulties of new species on reaching the termite hindgut successfully. Generally, the termite flagellate protist species are under strong co‐evolutionary constraints, and the major part of gut symbionts is endemic or native and probably co‐evolving with termite species (Hongoh et al., 2005; Noda et al., 2007). Evolutionary constraints may be closely linked with the specificity of the role of each flagellate protist in the different phases of the lignocellulose digestion process, which also dictates an environmental or functional filtering (meaning that only one species, or group of species, is able to occupy a certain ecological niche), as there exists a division of labor during the lignocellulose digestion process (Duarte, Duarte et al., 2017; Inoue, Murashima, Azuma, Sugimoto, & Slaytor, 1997; Raychoudhury et al., 2013; Todaka et al., 2007; Yoshimura, Fujino, Tsunoda, & Takahashi, 1996). Furthermore, the existence of a core group seems to be crucial for the maintenance of the termite holobiont, while other species may vary in function of the environment. Some social insects have the potential to became successful invasive species, relying on their ability to compensate for eventual bottleneck effects, resulting from the introduction events, through three different levels: population, colony (social traits), and individual (Ugelvig & Cremer, 2012). We would like to put forward a potential fourth level: the symbiotic fauna, expanding the gene pool available for adaptation. Through the synergy of combined abilities, symbionts can find faster solutions than individual organisms, and variability—the raw material for evolution—can arise from changes in either the host or the symbiont microbiome resulting in higher adaptation capacity and thus also higher invasiveness. Research providing data that confirms this statement should be envisaged, with other species and distribution ranges.

Regarding the aggressiveness testing, the aggressiveness index used seemed to need some adjustments, as the values for aggressive termites were consistently low, even though the mortality associated with those behaviors was considered to be high. As the index used was from testing with *Coptotermes formosanus* Shiraki termites (Husseneder & Grace, 2001), we suggest that, in the future, aggressiveness indices should be adjusted to termite species level. Confirming our last prediction, termites from mainland populations were shown to be more aggressive among neighboring colonies, unlike the introduced colonies from Horta city, which were not aggressive between themselves. This change of behavior, together with the observed high protist biodiversity, raises the hypothesis of an alternative mode of social organization of the Horta city colonies (when comparing with the endemic mainland populations). Low or no aggressive behavior among termites usually indicates that they recognize themselves as belonging to the same colony or that termites are organized into an extended colony model. This may be due to the loss of genetic diversity, probably resulting from the introduction event(s), in which the variability of termite's chemical signatures is reduced; therefore, the termites recognize all individuals as belonging to the same colony, as observed in France with the introduced *R. flavipes* termites (Perdereau et al., 2011). Additionally, the results obtained for community structures in Horta colonies, which were similar and among the most diverse observed in the whole study, may also contribute to the above hypothesis. The loss of aggressive behavior will favor the free contact among all individuals, as well as facilitating the allocation of food resources (Ugelvig & Cremer, 2012). This strategy may also promote the horizontal transmission of the symbionts among all the individuals, contributing to a more diverse pool of species of flagellate protists. For another subterranean termite (*Reticulitermes speratus* Holmgren), it was hypothesized that the intestinal bacteria might play an important role in the process of nestmate recognition (Matsuura, 2001).

The lack of aggressive behavior among termites has been also registered for an insular higher termite (Fuller, Jeyasingh, & Harris, 2004) and an invasive subterranean termite (Perdereau et al., 2011). The dear enemy phenomenon may be an explanation (Fisher, 1954; Temeles, 1994); however, this is not very common among subterranean termites, although Nobre, Nunes, and Bignell (2008) found different colonies of *R. grassei* foraging in the same resource at the same time. Along the distribution range of *R. grassei*, some authors detected a trend from the southern populations, with higher numbers of single‐family colonies, to the northern populations, exhibiting higher numbers of extended family colonies (DeHeer, Kutnik, Vargo, & Bagnères, 2005; Vargo & Husseneder, 2009; Vargo et al., 2013). The invasive *R. grassei* termites could have been introduced from different origins (meaning also different types of social structure), as this termite species is widespread throughout the Iberian Peninsula and Southwestern France (Bankhead‐Dronnet, Perdereau, Kutnik, Dupont, & Bagnères, 2015). The tendency of colonies to fuse into extended or mixed families may be influenced by the genetic bottlenecks resulting from the introduction event(s), which may lead to a homogenization of the chemical recognition cues and resulting lack of aggression between colonies (Perdereau et al., 2015; Suarez, Holway, & Tsutsui, 2008; Tsutsui, Suarez, Holway, & Case, 2000). This tendency may also emerge as a real need to survive in a new habitat, devoting all their energy to foraging and decreasing the costs of aggressive behaviors (Perdereau et al., 2015). Another factor which may have influenced the changing (or maintenance, if the introduced termites belonged to colonies organized into mixed or extended family colonies) of the invasive termite social structure is the fact that the Azorean islands are less seasonal environments, compared to mainland meteorological conditions, and *R. grassei* shows higher inbreeding in less seasonal environments (Vargo et al., 2013).

The termite symbiotic flagellate protist community seems to be composed of a core group of flagellate protists and a more flexible secondary symbiont community, all contributing to different loads to host fitness. The *Reticulitermes* ability to maintain its symbiotic flagellate protists diversity is a clear advantage as it will certainly be a valuable tool for the adaptation to different environments. As a successful invasive subterranean termite, it is easily introduced into new habitats by human‐mediated actions, and it is difficult to control, as the previous detection in UK and further tentative control measures show (Evans, Forschler, & Grace, 2013; Jenkins et al., 2001; Verkerk & Bravery, 2001). The socioeconomic and environmental impacts of this alien invasive species in the Faial Island are of high concern. Our results indicate that the Azorean *R. grassei* are organized into an extended or mixed colony model of social structure, either by the switching of social structure model from the native population, or because the native population was already organized in that social structure model, which has implications in the pest control management.

## CONFLICT OF INTEREST

None declared.

## AUTHOR CONTRIBUTION

S. Duarte was responsible for all major areas of concept formation, data collection, and analysis, as well as for writing the entire draft version of the manuscript and revising it according to coauthors’ comments. P.A.V. Borges and T. Nobre were involved in the concept formation and contributed to the manuscript review. L. Nunes was the supervisory author on this study and involved throughout it in concept formation and manuscript reviewing.
