# Supplementary material for: Symbiotic flagellate protists as cryptic drivers of adaptation and invasiveness of the subterranean termite Reticulitermes grassei Clément
Source: Ecol Evol. 2018 May 10;8(11):5242–53. doi: 10.1002/ece3.3819 (PMC6010709; doi:10.1002/ece3.3819)
Supplement: Supplementary file 1 [file ECE3-8-5242-s001.pdf]

Supplementary table. Average number of flagellate protists, per microliter of hindgut fluid, identified to morphotypes (n1 to n9, n13, n17 and n18) in each sampling point of Portugal geographical regions (AZ, BA, BE, EV, LE, PO, SA and SE).

		Code	n1	n2	n3	n4	n5	n6	n7	n8	n9	n13	n17	n18
**Azores (AZ)**	Horta	hor1*	939	2426	3061	1222	800	945	1120	2489	1396	835	800	995
	Horta	hor2*	1005	2810	2142	855	1113	1082	862	2400	1042	870	800	877
**Bragança (BA)**	Mirandela	mir*	965	9616	3008	949	-	857	800	2232	1666	-	800	-
	Bornes	bor	983	8312	1913	914	1013	867	974	4051	1505	859	-	865
**Beja (BE)**	Mértola	mer*	1303	8944	2338	-	-	-	864	4808	1469	-	-	-
	Moreanes	mor	1183	9712	4162	960	-	926	857	6476	2020	873	1029	933
**Évora (EV)**	Évora (city)	evc*	996	3255	3875	-	-	-	926	4767	2458	-	-	1000
	Évora (rural)	evf	1558	11800	3265	930	-	1097	850	6861	1870	800	800	-
**Leiria (LE)**	Alcobaça	alc*	1004	9800	2858	939	1162	853	867	6424	1173	926	-	-
	Nazaré	naz	1180	11472	2181	1150	1484	1013	1029	4702	1465	800	-	-
**Porto (PO)**	Porto	por*	1061	7814	3693	925	-	933	914	3564	1527	843	-	980
	Baltar	bal	1107	9544	2678	867	1247	840	1088	4718	1590	911	-	992
**Santarém (SA)**	Ourém	our*	1114	8488	2920	894	1012	800	880	3767	1479	1000	910	-
	Porto de Mós	mos	1232	9504	4726	900	919	883	889	5424	1618	923	-	-
**Setúbal (SE)**	Almada	alm*	995	10552	2763	933	912	800	800	5456	1125	873	-	-
	Sesimbra	ses	1050	4727	3000	800	800	920	985	6862	1128	892	864	800

Note: Codes followed by an asterisk refer to urban sites, the remaining sampling points refer to rural sites
